# Dietary Cannabidiol Supplementation on Growth Performance, Behavior, Blood Profile, Metabolomic Analysis and Fatty Acid Composition in Rabbits: A Multi-Disciplinary Approach to Improve Welfare and Productivity

**DOI:** 10.3390/vetsci12080759

**Published:** 2025-08-14

**Authors:** Nicola Francesco Addeo, Valeria Iervolino, Ruggero Amato, Mariarosaria Lanzieri, Daria Lotito, Maria Vittoria Tignani, Alessia Staropoli, Sara Damiano, Pietro Lombardi, Francesco Vinale, Giuliana Parisi, Fulvia Bovera, Nadia Musco, Vincenzo Mastellone

**Affiliations:** 1 Department of Veterinary Medicine and Animal Production, University of Naples Federico II, 80137 Naples, Italy; nicolafrancesco.addeo@unina.it (N.F.A.); valeria.iervolino@unina.it (V.I.); ruggero.amato@unina.it (R.A.); mariariosaria.lanzieri@unina.it (M.L.); daria.lotito@unina.it (D.L.); alessia.staropoli@unina.it (A.S.); sara.damiano@unina.it (S.D.); frvinale@unina.it (F.V.); fulvia.bovera@unina.it (F.B.); nadia.musco@unina.it (N.M.); vincenzo.mastellone@unina.it (V.M.); 2 Department of Agriculture, Food, Environment and Forestry (DAGRI), University of Florence, Via delle Cascine 5, 50144 Florence, Italy; mariavittoria.tignani@unifi.it (M.V.T.); giuliana.parisi@unifi.it (G.P.)

**Keywords:** oxidative status, metabolome profile, cannabidiol, rabbits, animal behavior, fatty acids

## Abstract

This study evaluated the effects of dietary cannabidiol (CBD) supplementation on behavior, blood parameters, oxidative status, metabolomic profile and the fatty acid composition of meat and liver in rabbits. A total of 42 New Zealand White × California rabbits (60 days old; 1:1 sex ratio; average weight 1621.3 ± 46.2 g) were randomly assigned to two groups (a control group, CTRL, and a CBD group, n = 21 each). Both groups received the same commercial diet, with the CBD group additionally supplemented with 0.1 mL of cannabis extract in coconut oil, corresponding to 10 mg CBD/animal/day. At 92 days of age, rabbits were slaughtered, and samples were collected for analyses. Results showed that CBD supplementation significantly improved body weight gain, reduced plasma triglyceride levels, and enhanced oxidative status. Behavioral observations indicated increased motor and grooming activities in CBD-supplemented animals, suggesting enhanced psychological well-being. The fatty acid profile of meat and liver was not significantly altered by CBD supplementation. Overall, dietary CBD demonstrated the potential to positively influence physiological and behavioral responses, representing a promising strategy to enhance animal welfare and productivity in rabbit farming. Although no adverse effects on lipid profiles were observed, further studies are warranted to explore CBD’s role in lipid metabolism and cholesterol regulation.

## Simple Summary

Animal health and welfare are essential for ethical farming and high-quality food production. This study evaluated the effects of dietary cannabidiol (CBD) supplementation on behavior, some blood parameters, and fatty acid composition in meat and liver of rabbits. CBD is gaining attention for its pharmacological properties and its role in the endocannabinoid system. The results suggest that CBD supplementation can influence behavioral and physiological responses in rabbits, offering potential benefits for both animal welfare and meat quality.

## 1. Introduction

Rabbit farming offers a more sustainable alternative to traditional livestock production with lower environmental impacts and superior nutritional benefits, aligning with the 2030 Sustainable Development Goals [1,2,3]. Additionally, rabbit meat is highly digestible and can be considered a functional food with lower fat, cholesterol, and saturated fatty acid content compared to other meats, making it a healthier dietary choice [4]. This combination of efficient production and nutritional value of the meat reinforces the role of rabbit farming in promoting sustainable and health-conscious food systems. To optimize rabbit breeding, there has been a significant evolution in feeding techniques for rabbits, in recent years. The use of additives in animal feed has always attracted interest in scientific research, aiming to enhance the health and well-being of farmed animals [5]. In this context, hemp (*Cannabis sativa* L.) is emerging as a dietary ingredient and functional additive for livestock feeding, due to its rich nutritional profile and potential to improve animal health and product quality [6]. There are more than 90 known cannabinoids [7]; among them, two are of particular interest: delta-9-tetrahydrocannabinol (THC) and cannabidiol (CBD) [8]. THC is a psychoactive molecule with both therapeutic and recreational applications, but its intoxicating properties necessitate strict regulation [9,10]. Conversely, CBD is non-psychoactive, has a favorable safety profile, and offers a range of therapeutic benefits without intoxication [10,11]. CBD, primarily derived from hemp flowers, leaves, and stems, is in high demand in the medical, pharmaceutical, and nutraceutical industries [5]. It exhibits anti-inflammatory, antioxidant, analgesic, antinociceptive, anxiolytic, antipsychotic, neuroprotective, appetite-stimulating, and anticonvulsant effects [12,13,14]. CBD-based products are also gaining popularity in veterinary medicine, particularly for companion animals [15,16]. CBD’s specific effects in livestock remain underexplored, although some evidence of hemp benefits has now been further investigated, even in animal husbandry [16,17,18]. Nonetheless, the action of CBD is mainly linked to its interaction with the endocannabinoid system (ECS), present in almost all animal species [19], which plays a crucial role in the regulation of physiological functions such as immune response and pain perception [8]. By modulating ECS, CBD can promote calmness and relaxation and help cope with stressful situations [20]. This has led to a growing interest in its application as a feed supplement in farm animals. This combined action of CBD on anxiety and gastrointestinal function may be particularly beneficial in rabbits (*Oryctolagus cuniculus*), commonly bred in cages for meat production [21]. 

Although several studies have been conducted on the use of hemp in rabbit nutrition [22,23,24] and on the effect of CBD on humans and other animal species, limited research has been carried out on CBD application in rabbit diets. Preliminary data suggest that CBD may positively influence various physiological parameters, such as the lipid profile, reducing triglyceride and cholesterol levels [25,26]. Rabbits are known to be particularly sensitive to stress, especially in intensive farming environments, where cages can limit the expression of their natural behaviors, raising ethical concerns about their welfare [27]. Although various cage and housing systems have been proposed to compare the level of welfare [28], rabbits remain significantly susceptible to stress due to their inability to fully express their innate behaviors. The introduction of dietary interventions such as those based on CBD could represent an effective strategy to mitigate the negative effects of environmental stress and improve the animal’s quality of life. Therefore, investigating the effect of CBD on this species could provide useful information to improve both nutritional management and the overall welfare of these animals.

The present study aimed to investigate the effects of dietary CBD supplementation on growth performance and meat quality of rabbits in the period of 65–92 days of age. At the same time, the potential of CBD to improve the general health of rabbits was explored by investigating blood biochemistry and metabolomics, oxidative stress, behavior, and fatty acid profile of the meat and liver, taking into account the possible gender differences.

## 2. Materials and Methods

### 2.1. Animals and Diet

The University of Napoli Federico II Ethical Animal Care and Use Committee in Italy approved all experimental protocols (protocol number 2019/0058989). The study complied with the EC Directive 2010/63/EU [29], which outlines the protection of animals used for scientific purposes.

A total of 42 New Zealand White × California rabbits, aged 60 days, with a balanced sex ratio (1:1) and an average weight of 1621.3 ± 46.2 g (measured using a Digital Rabbit Scale, model WS20, OLBA B.V., Coevorden, Netherlands), were systematically allocated into two groups, each consisting of 21 animals. The rabbits were housed individually in cages measuring 25 cm in length, 45 cm in depth, and 30 cm in height within a controlled environment (temperature 21 °C, relative humidity 62%, 16 h light/8 h dark photoperiod). During the acclimation period (from 60 to 64 days of age), all rabbits consumed an identical commercial diet. Beginning at 65 days of age, rabbits were divided in two groups, both fed this basal diet; however, the group receiving cannabidiol (CBD) supplementation (comprising 11 females and 10 males) was additionally administered 0.1 mL of a cannabis extract in coconut-based oil (Giantec; Isernia, Italy), equating to a daily dose of 10 mg of CBD per animal. The dosage was determined based on existing literature on laboratory rabbits [30] and manufacturer recommendations. The cannabis extract was delivered individually by applying it to a 15 g alfalfa-dehydrated meal-based wafer, which was consumed entirely by each rabbit. Conversely, the control group (CTRL; 10 females and 11 males) received the same alfalfa wafer with 0.1 mL of coconut oil but without the cannabis extract. From 65 to 92 days of age (for a total of 27 days), individual body weight and feed intake were monitored on a weekly basis. Feed intake was determined for each rabbit by weighing the feed offered and the residual feed, using individual hopper feeders designed to minimize feed losses.

The CBD extract was analyzed via High Performance Liquid Chromatography with a Diode Array Detector (HPLC-DAD), in accordance with the European Pharmacopoeia (Ph. Eur. 2.2.29) [31], and the analytical results are detailed in Table 1. 

The composition and chemical characteristics of both the diet and the wafer were sourced from commercial labels and are provided in Table 2. The ingredients were obtained from the commercial tag, and the chemical characteristics were determined according to AOAC (2002) [32] official methods to determine dry matter (DM, ID 934.01), the composition of ether extract (EE, ID 920.39), crude protein (CP, ID 2001.11), crude fiber (CF, ID 978.10) and ashes (ID 942.05). The fatty acid profile of CBD oil and the diet were analyzed as described in Section 2.4. and are given in Table 3 and Table 4, respectively. 

The digestible energy (DE) content was estimated using the equation proposed by Fernandez-Carmona et al. (1996) [33]:

DE (MJ kg−1 DM) = 14.2 − 0.205 Acid Detergent Fiber (ADF) + 0.218 Ether Extract (EE) + 0.057 Crude Protein (CP) (R^2^ = 0.965; Relative Standard Deviation, RSD = 0.494).

At 92 days of age, eight rabbits from each group (maintaining a 1:1 sex ratio) were transported to a specialized slaughterhouse. The protocol for slaughter, processing and analysis of carcass was done according to Hassan et al. (2016) [34]. The pelt, tail, and visceral parts were removed after the complete draining of the blood, and the remaining parts were designated as carcasses. The animals were kept under observation for approximately 2 h before slaughter, with access to water but without feed. After slaughtering, the carcasses were weighed and then refrigerated at 4 °C for 24 h in a ventilated room. After 24 h of refrigeration, carcasses were weighed again to obtain the refrigerated carcass weight (CC); then, skin, head, liver, heart, lungs + esophagus + trachea + thymus gland bundle, spleen, kidneys, and reproductive traits were removed and weighed individually (Sigma-Aldrich electronic balance, model Z742838, Merck KGaA, Darmstadt, Germany) to obtain the reference carcass (RC). Using a portable instrument (Model HI 9025; Hanna Instruments, Woonsocket, RI, USA), equipped with the electrode FC 230C (Hanna Instruments), the pH value was measured in the *Biceps femoris* (BF) and *Longissimus dorsi* (LD) muscles, 45 min and 24 h after slaughter. 

### 2.2. Behavioral Analysis

Video recordings lasting 90 min each were conducted four times every seven days. The recording periods (12:00–15:00) were consistent across all weeks and were selected after preliminary observations, which characterized the periods of greatest daytime activity of animals. Also, the caring procedures started after the end of recording and, throughout the recording periods, measures were implemented to ensure that no individuals had access to the animals, preventing any interference with their usual behavioral patterns. The footage was captured using four AP-320S (C&Xanadu, Shanghai, China) cameras strategically positioned in the corners of the cages. The recordings for each group were watched and coded by an expert observer. A second operator coded 20% of the videos, and the interobserver reliability was found to be very high for all observed behaviors, ranging from 90 to 96%. Behavior was categorized according to the following ethogram: grooming, moving, setting, lying, looking around, gnawing, stretching and sniffing. For each animal, the duration of the observed behaviors was measured. 

### 2.3. Blood Biochemistry and Oxidative Status 

At the end of the experiment, blood samples were collected into tubes with and without K3-EDTA, and immediately transported to the laboratory. Serum was obtained by centrifugation at 1200× *g* for 15 min, then divided into aliquots and frozen at −80 °C.

Blood chemistry analyses on serum aliquots were performed by an automatic biochemical analyzer (AMS Autolab, Diamond Diagnostics, Holliston, MA, USA), using reagents from Spinreact (Girona, Spain) to determine: total proteins (TP), albumin (ALB), creatinine (CREA), glucose (GLU), aspartate amino transferase (AST), alanine-aminotransferase (ALT), gamma-glutamyltransferase (GGT), bilirubin (BIL), alkaline phosphatase (ALP), cholesterol (CHOL), and triglycerides (TRI). Reactive oxygen metabolites and the biological antioxidant potential were performed on serum aliquots using reagents from Diacron International s.r.l. (Grosseto, Italy).

### 2.4. Metabolomic Analysis

A quantity of 50 µL of rabbit’s serum was mixed with 150 μL of methanol:chloroform 3/1 *v*/*v*. The mix was sonicated with an ultrasonic bath (Sonorex, Bandelin Electronic GmbH & Co. K, Berlin, Germany) for 5 min and then left at −20 °C for 10 min for protein precipitation. The samples were centrifuged at 12,000 rpm for 30 min at 4 °C and the supernatant was recovered and dried under a gentle nitrogen flow. The crude extracts were resuspended in 100 µL of N-Hexane and derivatized with 100 µL of N,O bis(trimethylsilyl)trifluoroacetamide (BSTFA, Merck, Darmstadt, Germany), in an ultrasonic bath for 30 min. The trimethylsilyl derivatives were analyzed using an Agilent 8890 GC instrument (Milan, Italy) linked to an Agilent 5977B Inert MS. A HP-5MS capillary column ((5%-phenyl)-methylpolysiloxane stationary phase) was employed for the separation. The following oven GC temperature program was used: initial temperature of 80 °C for 2 min, rising at 10 °C/min to 180 °C; rising at 5 °C/min to 240 °C; rising at 25 °C/min to 290 °C and holding for 9 min. The solvent delay time was set at 5 min. The GC injector was set to splitless mode at 270 °C and the carrier gas was helium, at a flow rate of 1 mL/min; the transfer line temperature was set at 220 °C. Measurements were carried out in full scan mode (*m*/*z* 30–550) with electron impact (EI) ionization (70 eV). The temperatures of the EI ion source and the quadrupole mass filter were fixed at 260 and 150 °C, respectively. Identification of metabolites was obtained by comparison of deconvoluted mass spectra collected in NIST 20 library of known compounds (National Institute of Standards and Technology).

The metabolomic analysis was carried out on 8 biological replicates and each analysis was performed 3 times (technical replicates). Serum samples representing a biological replicate were collected from randomly selected animals. 

### 2.5. Total Lipids and Fatty Acid Profiles of Feedstuff, Meat Cuts, and Livers

Total lipids from diets, meat cuts (thighs and loins) and liver were quantified gravimetrically. This was performed by extracting 2 g of each sample using the method described by Folch et al. (1957) [35]. The lipid extracts were then converted into fatty acid methyl esters (FAME) through base-catalyzed trans-esterification, following the procedure outlined by Christie (1982) [36].

The fatty acid composition of the CBD extract, dietary samples, meat and liver was analyzed using a Varian GC 430 gas chromatograph (Varian Inc., Palo Alto, CA, USA), equipped with a Supelco Omegawax™ 320 capillary column (Supelco, Bellefonte, PA, USA) and a flame ionization detector. An aliquot of 1 μL of lipid extracted sample, dissolved in hexane, was injected in GC, with helium serving as the carrier gas at a constant flow rate of 1.5 mL/min and a split ratio of 1:20. The gas chromatograph (GC) oven was initially held at 120 °C for 0.5 min, then ramped to 170 °C at a rate of 10 °C/min over 6 min, and subsequently to 220 °C at a rate of 3 °C/min over 12 min, where it was maintained for 13 min, resulting in a total runtime of 40 min. Injector and detector temperatures were set at 220 and 300 °C, respectively.

Fatty acids were identified by comparing FAME retention times to those of a Supelco 37-component FAME mix standard (Supelco, Bellefonte, PA, USA), and quantified using calibration curves with tricosanoic acid (C23:0) (Sigma-Aldrich, St. Louis, MO, USA) as internal standard. Chromatogram recordings were conducted with the Galaxie Chromatography Data System version 1.9.302.952 (Varian Inc., Palo Alto, CA, USA).

### 2.6. Cholesterol Content of the Loins, Thighs, and Livers

Cholesterol content was measured following the procedure described by Secci et al. (2018) [37]. In brief, 0.2 mL of lipid extract was mixed with 0.5 mL of 5α-cholestane (0.2 mg/mL in chloroform) as internal standard (Supelco, Bellefonte, PA, USA). After evaporating the solvent, 5 mL KOH (0.5 M in methanol) was added, and the mixture was then saponified by heating in a water bath at 95 °C for 40 min. Following saponification, 3 mL of distilled water and 2 mL of n-hexane were added, and the upper phase was collected for the gas chromatography run. The analysis was performed on a Varian GC 430 gas chromatograph (Varian Inc., Palo Alto, CA, USA), equipped with a flame ionization detector and a Supelco SACTM fused silica capillary column (30 m × 0.25 mm i.d., 0.25-μm film; Supelco, Agilent Technologies, Santa Clara, CA, USA). An aliquot of 1 µL was injected with a 1:100 split ratio at 300 °C. The oven temperature was planned to increase from 130 to 290 °C at a rate of 20 °C/min over 8 min and then maintained at 290 °C for 11 min. The detector temperature was set at 300 °C, with helium as the carrier gas, at a constant flow rate of 1.3 mL/min.

### 2.7. Oxidative Status of the Loins, Thighs, and Livers 

To assess the primary metabolites formed from lipid oxidation in rabbits’ loins, thighs, and livers, conjugated dienes (CD) were quantified, using 0.5 µL of lipid extract dissolved in 3 mL of pure hexane, as described by Srinivasan et al. (1996) [38]. Absorbance was measured at 232 nm with a 50 Scan spectrophotometer using Cary Win UV software version 5.3, and the concentration of hydroperoxides (mmol/kg) was calculated based on a molar extinction coefficient of 29,000 mL/mmol × cm. Lipid peroxidation in meat was further evaluated through malondialdehyde (MDA) levels. The secondary oxidative products in the meat and liver samples were assessed as thiobarbituric acid reactive substances (TBARS), using a colorimetric method adapted from Secci et al. (2016) [39]. The procedure involved dissolving the sample in 5% trichloroacetic acid (TCA) and adding 0.02 M thiobarbituric acid (TBA). After incubating at 97 °C for 40 min, the oxidation products were quantified by comparing the absorbance at 532 nm to calibration curves prepared with 1,1,3,3-tetraethoxypropane (TEP) in 5% TCA, with concentrations ranging from 0.2 to 3.1 mmol/L. The TBARS results were expressed as mg of malondialdehyde equivalents (MDA eq.) per 100 g of sample.

### 2.8. Statistical Analysis

Data were processed with a two-way ANOVA by using the GLM procedure of SAS (2002) [40], according to the following model:

Yijk = m + Gi + Sj + GxSij + eijk

where Y is the single observation, m the general mean, G the effect of the group differently fed (i = CTRL or CBD), S the effect of the gender (j = female or male), G × S the interaction between the two main factors, and e the error. 

Before data analysis, the normal distribution of the data was checked [40]. Comparison among means was performed by Tukey’s test [40] at *p* < 0.05. *p* values lower than 0.05 are considered significant, while *p* values ranging between 0.05 and 1 are considered a tendency.

Statistical analysis of metabolomic data was performed by Mass Profile Professional Software Version 13.1.1 (Agilent Technologies). Row data were aligned, normalized and grouped by the dietary treatment (CTRL or CBD) administered to rabbits and then subjected to Principal Component Analysis (PCA) and hierarchical clustering analysis, to look for trends between and within the two conditions. Student’s *t*-test (*p* < 0.05) and fold change (>2.0) were used to compare the metabolomic profiles of rabbits in CTRL and CBD groups. Metabolites were finally identified by comparison of deconvoluted mass spectra collected in NIST (National Institute of Standards and Technology) 20 library of known compounds.

## 3. Results

Chemical analysis of the cannabis extract showed that the total amount of cannabidiol (CBD + CBDA) was the highest active ingredient of the product (10.35%), while very low levels (0.19%) of total tetrahydrocannabinol (THC + THCA) were detected (as shown in Table 1). 

Table 3 shows the main fatty acids detected in the lipid profile of the CBD extract used in this study, predominantly composed of saturated fatty acids (SFAs), accounting for nearly 84 g FA/100 g of the total FAME. Among unsaturated fatty acids, n-6 PUFAs, primarily represented by linoleic acid (C18:2n-6), and n-3 PUFAs, with α-linolenic acid (C18:3n-3) as the most abundant, were present in smaller proportions.

Regarding the chemical-nutritional characteristics and ingredients of the basal diet and wafer used along the trial, in Table 2 the proximate composition, the content in Ca, *p* and Na, in some amino acids (lysine and methionine), and the value of digestible energy are reported while the fatty acid profile of the diet can be found in Table 4. In the diet, n-6 PUFAs emerged as the predominant fatty acid class, followed by MUFAs, SFAs, and n-3 PUFAs. 

### 3.1. Growth Performance and Carcass Characteristics 

Regarding the in vivo results (Table 5), the live weight (LW) of the rabbits was significantly affected by CBD supplementation at the end of the experiment (*p* = 0.05), according to a significant (*p* < 0.01) increase in body weight (BWG) recorded in the CBD group along the experimental period (from 65 to 92 days) compared to the CTRL group. Considering the whole experimental period, the difference in feed intake (FI) between sexes was statistically significant, with the highest values in males (*p* < 0.01). In the experiment carried out, daily feed consumption showed a non-linear trend, recording an average consumption of about 160 g/rabbit/day in both groups. The ability of the cannabinoid system to control appetite, feed intake, and energy balance has recently received much attention, especially in light of the different modes of action underlying these functions. Feed conversion ratio (FCR) was significantly (*p* < 0.01) lower in the CBD group during the whole experimental period. No interaction effects between group and sex were recorded.

Table 6 shows the characteristics of rabbit carcasses. Warm carcass weights and carcass yield were significantly higher (*p* < 0.05 and *p* < 0.01, respectively) in the CBD group compared to the CTRL one. Spleen, as a % of warm carcass, was heavier in the CBD group while liver was heavier in the CTRL group (*p* < 0.01). Baseline carcass weight was higher in the CBD group (*p* < 0.05), while inguinal fat was significantly (*p* < 0.01) heavier in the CTRL group. Our results indicated a significant difference related to sex in in vivo performance, particularly in terms of body weight gain (BWG), males showing higher BWG at 65–70 days (*p* < 0.05) compared to females. However, in the period 86–92 days, BWG was lower in males (*p* < 0.01), with no significant differences observed over the overall trial period (65–92 days). Additionally, no significant disparities were found between males and females in post-mortem outcomes, except for carcass yield, reference carcass weight and warm carcass weight being higher in males (*p* < 0.01, *p* < 0.05 and *p* < 0.05, respectively) and perineal fat (in %) being higher in females (*p* < 0.05). No interaction effects between group and sex were recorded.

### 3.2. Behavioral Analysis 

The study assessed the behavior of rabbits by quantifying the duration of various activities. In Table 7, in terms of resting behavior, the CTRL rabbits spent more time lying down (7458.2 s) than the CBD rabbits (5875.6 s), although this difference did not achieve statistical significance (*p* = 0.0850).

In Table 8, during the initial phase of the experiment (day 9 of the trial), rabbits fed the CBD diet demonstrated a statistically significant increase in time spent on locomotor activities (Moving; *p* = 0.0192).

At the end of the trial (Table 9), rabbits treated with CBD exhibited a significant (*p* = 0.0498) increase in grooming behavior (1820.5 s) compared to the other group (1019.2 s). Although the difference in movement was not statistically significant, there was a tendency for increased movement in the CBD group (101.03 vs. 33.40 s).

**Figure 1:**
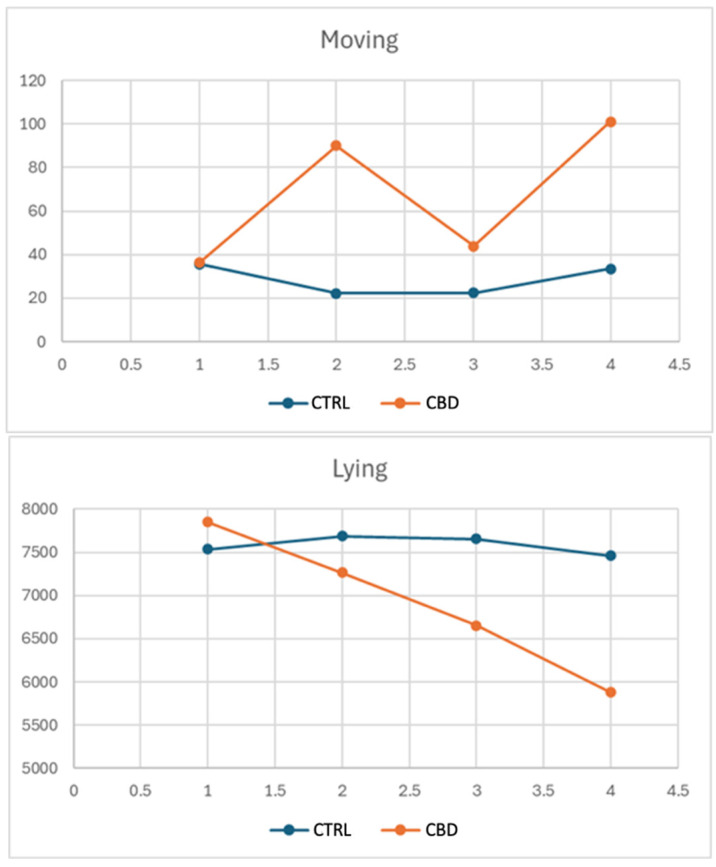
Trend of moving and lying behaviors along the trial.

Figure 1 illustrates the effects of the diet administered on two distinct behaviors: movement and time spent lying down. In particular, the CBD group showed a marked increase in movement, with values reaching approximately 100 units at the second and fourth time points, compared to the CTRL group, which remained stable between 20 and 40 units. Similarly, the time spent lying down decreased significantly in the CBD group, dropping from values similar to those of the CTRL at the beginning to around 5500 units by the fourth time point, while the CTRL group remained stable at about 7500–7800 units. 

### 3.3. Blood Profiles 

The study assessed the blood profiles of rabbits based on dietary treatment, gender, and the interaction between these factors (Table 10). 

Based on the results of the blood profile, a statistically significant reduction in triglyceride levels (*p* = 0.0217) was found in rabbits treated with CBD. Additionally, the albumin/globulin ratio exhibited a significant decrease in the CBD-treated group (*p* = 0.0221), as for the cholesterol levels in the same group, their values tended to be lower compared to the CTRL one, with a *p*-value of 0.0853, suggesting a potential influence of diet on cholesterol levels. The levels of the derivates of reactive oxygen metabolites (dROMs) were significantly lower in the CBD group, indicating a reduced oxidative stress (*p* = 0.0022). Conversely, the biological antioxidant potential (BAP) values were higher in the CBD group, suggesting an enhanced antioxidant capacity (*p* = 0.003). The group receiving CBD demonstrated significantly lower triglyceride levels compared to the CTRL group (*p* = 0.0217). Furthermore, a significant difference in the albumin/globulin ratio was also highlighted between the different groups and between sexes, with a lower value in females. In particular, the CTRL group showed a higher albumin/globulin ratio than the CBD group (*p* = 0.0221).

### 3.4. Metabolomic Analysis

**Figure 2:**
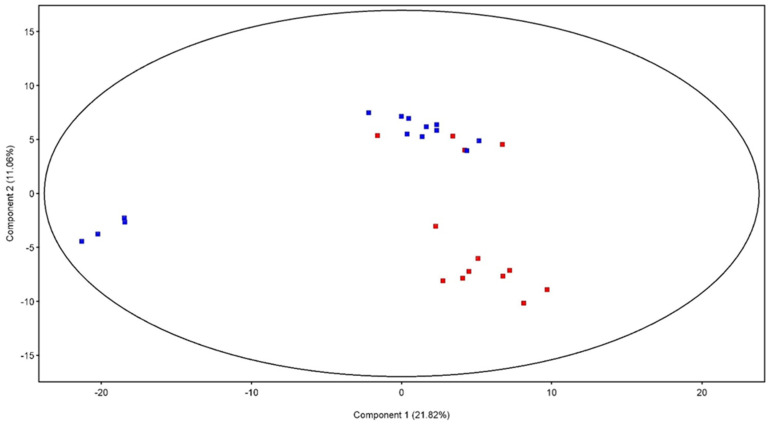
Two-dimensional principal component analysis (PCA) scores plot of the GC-MS dataset from rabbits’ serum. The different colors identified the groups, respectively: CTRL blue squares; CBD red squares. PC1 accounted for 21.82%, and PC2 accounted for 11.06% of the total variance.

**Figure 3:**
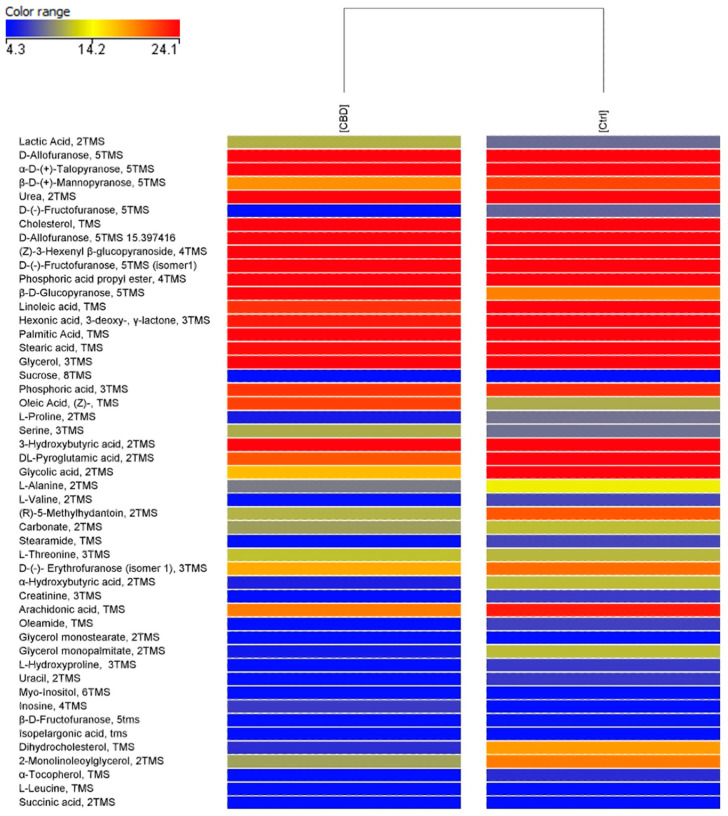
Heatmap obtained from comparison of metabolomic profiles of rabbit’s serum extracts. The range of colors indicates normalized intensity values ranging from blue (less abundant) to red (most abundant). TMS= Trimethylsilyl derivative.

PCA score plot (Figure 2) shows that the rabbit’s serum metabolome changed after the treatments, and a separation of samples in the second component of variance was registered. A minimum spread for the CTRL (blue) is noted, while for the treated group (CBD, in red) there is a spread with four subclasses of samples. In Figure 3, the hierarchical cluster obtained from the metabolomic profiles of the two groups is reported. By categorizing the data of each group, hierarchical cluster analysis characterizes similarities and differences in the metabolic profiles of the groups (CTRL, CBD). This figure represents the normalized abundance of each compound that is present or absent. The clustering clearly demonstrated a different accumulation of serum molecules between the two groups, suggesting that the metabolic profile was affected by the dietary treatment in a unique way.

Metabolomic analysis revealed 343 compounds as a result of the data processing. Among them, 35 differently accumulated compounds were highlighted by univariate statistical analysis (T-test, *p* < 0.05 and fold change > 2.0). Three significant compounds were identified and reported in Table 11, respectively, D-(-)-Fructofuranose, 5TMS (isomer 1); Dihydrocholesterol, TMS; and 2-Monolinoleoylglycerol, 2TMS. All the identified compounds are down-regulated by CBD’s treatment.

### 3.5. Total Lipids and Fatty Acid Profile of the Loins, Thighs, and Livers 

The complete fatty acid profiles in the thigh and loin of rabbits, and in their livers, were analyzed, resulting in limited significant effects of dietary treatment on individual fatty acid content. The total lipids and the fatty acid profile of the loins are reported in Table 12. In the loins, SFAs such as C10:0 and C12:0 were significantly higher in rabbits fed the CTRL compared to those fed the CBD-enriched diet. Female rabbits displayed higher concentrations of C17:0 than males which, conversely, exhibited higher levels of eicosadienoic acid (C20:2n-6) in the same cut.

Sex exerted a more pronounced influence on thigh fatty acid profiles (Table 13), since female rabbits contained higher levels of C17:0, as in the loins, and C18:0 fatty acids, whereas the thighs of male rabbits had a greater concentration of C20:2n-6, as in the other cuts. Interestingly, C20:1n-9 showed a trend toward being more abundant in the hind limb muscle of males, although this did not reach statistical significance (*p* = 0.074), as well as C10:0 and C12:0 (*p* = 0.060 and 0.087, respectively).

Rabbits consuming the CBD diet had livers with significantly higher concentrations of palmitoleic acid (C16:1n-7), as shown in Table 14. For the concentration of C20:0 fatty acid, a significant interaction between sex and dietary group was found. Additionally, eicosadienoic acid (C20:2n-6) and the n-6/n-3 ratio were significantly higher in male rabbits. 

**Figure 4:**
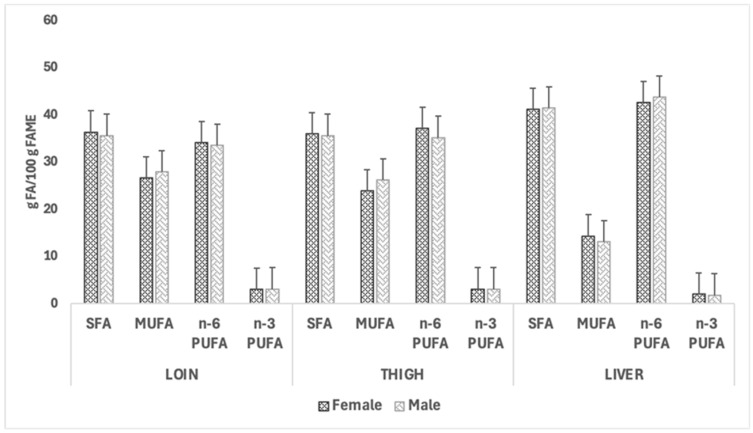
Effect of sex on the fatty acid class composition (g fatty acid/100 g fatty acid methyl esters) in loins, thighs, and livers of the female and male rabbits. Abbreviations: FA: saturated fatty acids, MUFA: monounsaturated fatty acids, PUFA: polyunsaturated fatty acids.

**Figure 5:**
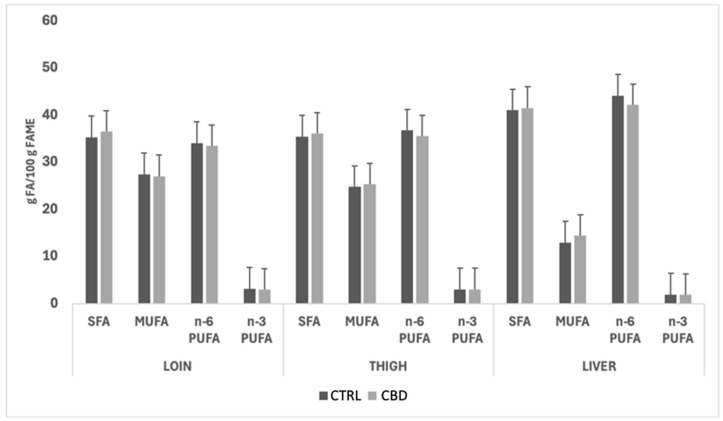
Effect of the diet on the fatty acid class composition (g fatty acid/100 g fatty acid methyl esters) of loins, thighs and livers of rabbits. Abbreviations: FA: saturated fatty acids, MUFA: monounsaturated fatty acids, PUFA: polyunsaturated fatty acids.

Figure 4 and Figure 5 illustrate the impact of sex and dietary treatment, respectively, on the fatty acid class composition of loins, thighs, and livers of CTRL and CBD groups. In both figures, no significant effects of the dietary treatment were observed for the fatty acid group composition; however, quantitatively, the most abundant fatty acid classes in both females and males were SFAs and n-6 PUFAs across the two muscles and the liver. The liver exhibited reduced MUFA levels compared to the muscle cuts, and the n-6 PUFA content was consistently higher in this organ. A similar pattern was observed when comparing the fatty acid group profiles in rabbits fed the two dietary treatments.

### 3.6. Cholesterol of Meat Cuts and Livers 

The cholesterol content did not exhibit significant differences across meat cuts or liver for any of the fixed effects (Table 15); however, a trend was observed for the interaction between diet group and sex in the loin (*p* = 0.077). Although no statistical differences were detected, the CBD-fed rabbits displayed a higher cholesterol content, not in the loin but slightly in the thigh and notably in the liver compared to the CTRL group.

### 3.7. Oxidative Status of Meat and Livers 

Regarding the lipid oxidative status in the loins and thighs, no significant effects on malondialdehyde (MDA) and conjugated dienes (CD) levels were observed related to diet, sex, or their interaction, thus indicating that the factors investigated did not have a significant impact on tissue lipid oxidation. The only exception was the level of conjugated dienes in the liver, which was significantly higher in the CTRL rabbits (43.35 vs. 25.96 mmol Hp/kg lipid).

## 4. Discussion

The results of this study indicated that the dietary supplementation of CBD in rabbit diets may have several physiological, productive and behavioral benefits and could represent a promising strategy for improving animal welfare and health management. At the end of the experimental period, the live weight of CBD-treated rabbits was higher than that of the CTRL group. In terms of feed intake, considering the entire duration of the experiment (65–92 days), our results agree with those of Horakova et al. (2020) [41] who found no significant differences between CBD and CTRL groups (*p* = 0.45). Despite this, considering the entire experimental period, feed intake showed significance for the interaction group × sex. As for the feed conversion ratio, Horakova et al. (2020) [41] showed a trend towards higher values in the experimental group treated with hemp herb, although without significant differences. In agreement with this observation, in the present study an improvement in the feed conversion ratio in the CBD-treated group during the whole experimental period was recorded. 

### 4.1. Growth Performance and Carcass Characteristics

Growth performance, however, showed significant differences between sexes. Between the 65th and 70th day of age, males showed a faster growth than females, whereas between the 86th and 92nd day, females had outpaced males in weight gain. This could indicate that there is a difference in the growth patterns of males and females, especially in the later stages of development [42]. Indeed, this observation is supported by Setiaji et al. (2013) [43], who found differences in growth patterns between sexes at specific stages of development. Based on what was described above, it can be stated that the reason for the discrepancies between our results and those of other studies could be connected to the different experimental conditions related to dietary treatment. Some studies carried out in other species reported that *Cannabis sativa* had no impact on carcass traits of sheep [44], goats [45], and chickens [46]. Conversely, when specifically utilizing cannabidiol as one of the non-psychoactive compounds of *Cannabis sativa*, it can increase carcass characteristics, as shown in our study, where the supplementation significantly increased warm carcass weight and carcass yield. Moreover, spleen weight as a percentage of the warm carcass was higher in the CBD group, probably due to the immunomodulatory effect of cannabinoids through the activation of selective receptors changing the activity of various immune cells [47]. The liver was heavier and the percentage of inguinal fat was significantly higher in the control group, suggesting that the administered CBD may have influenced fat deposition, reducing its accumulation in certain regions of the body. This is supported by studies conducted on other animal models, such as that of Erukainure et al. (2022) [26], in which CBD was also shown to have effects on lipid metabolism. Although few sex differences were observed, some results included a higher warm carcass weight, a higher reference carcass weight, and a higher carcass yield in males. In summary, this indicates that males provide more usable meat. However, perineal fat percentage was higher in females, which confirms a gender variation in fat distribution [48]. The results obtained in this trial indicate that CBD may be a new feed additive for rabbits, which helps to improve meat production without increasing feed intake. Long-term studies will be needed to fully understand the implications of CBD on rabbit health and the mechanisms underlying variations in growth performance. 

### 4.2. Metabolomic Analysis

An untargeted metabolomics approach [49] has been applied to compare the serum metabolomic profiles of the different groups and GC-MS datasets were first submitted to Principal Component Analysis (PCA, Figure 1) and shown as a hierarchical cluster (Figure 2). PCA score plot showed that serum metabolomic profiles were affected by the different dietary treatments, revealing a clear separation between the CTRL group and the CBD-treated group in the second component of variance. The clustering pattern suggests that CBD administration induced specific metabolome profile changes. Based on the hierarchical cluster, the CBD-treated group exhibited a marked shift in abundance compared to the CTRL group, probably due to specific lipid metabolism, amino acid turnover, and energy pathways. In particular, the untargeted metabolomic analysis revealed that dihydrocholesterol and 2-monolinoleoylglycerol were down-regulated in the CBDs group rabbits. The downregulation of dihydrocholesterol in serum obtained from animals treated with CBD suggests a lower susceptibility to diseases related to aorta plaques [50]. 2-Monolinoleoylglycerol, also known as 2-linoleoylglycerol is involved in anti-inflammatory effects [51] and regulatory roles in lipid metabolism [52]. Finally, the downregulation of fructofuranose indicates an impact on simple carbohydrate pathways. Accordingly, previous studies showed that CBD can affect energy balance and glucose regulation, with possible implications on bioenergetics and systemic metabolism [53].

### 4.3. Blood Profiles 

Additionally, the decreases in triglycerides in CBD group are in line with existing literature, which suggests that there is an involvement of CBD in lipid metabolic pathways [25], probably mediated by interaction with the endocannabinoid system. The hypotriglyceridemic effect can therefore be linked to the action of CBD in modulating the synthesis or use of triglycerides, as discussed by Dochez-Arnault et al. (2023) [25], due to the inhibition of lipogenesis or due to the enhancement of fatty acid beta-oxidation, as reported in a previous paper [54]. Regarding cholesterol, a trend was observed in the CBD group, with lower cholesterol levels than the CTRL group. This highlighted that CBD may have a beneficial potential for lipid metabolism, as confirmed by Erukainure et al. (2021) [55]. Moreover, regarding blood profiles, the ratio Alb/Glob indeed yielded a significantly different between the two groups, with a lower value in the CBD group. It follows that CBD could act as a modulator of the balance of the two proteins in the blood and is therefore relevant to health regarding protein and immune function [56]. In fact, an imbalance in this ratio can be associated with inflammatory conditions, as documented by Cattaneo et al. (2021) [57]. These results indicate that CBD could have a promising role in the management of conditions related to immune dysfunction or alterations in protein balance, broadening the potential therapeutic applications of this compound. The well-documented antioxidant and anti-inflammatory action of CBD [58,59,60] further underlines its role in improving animal health. This significant decrease in dROMs observed in this study supports the antioxidant efficacy of CBD which could be due to an imbalance between the production of reactive oxygen species (ROS) and antioxidant defense systems [61]. Such an imbalance can compromise many important physiological functions, such as nutrient absorption and digestion, making animals more susceptible to disease exposure [62]. Thus, CBD can modulate the oxidative balance by changing the levels and activities of both antioxidants and pro-oxidants independently of classic cannabinoid receptors [63]. It has been documented that CBD improves the activity of antioxidant enzymes, while also stopping free radical chain reactions [64,65,66]. This could be especially beneficial for rabbits because they are susceptible to oxidative stress due to environmental conditions and dietary challenges. Therefore, the improvement in Biological Antioxidant Potential (BAP) in CBD-treated groups may be indicative of an increase in endogenous antioxidant capacity, which can counteract cell damage induced by ROS, thus significantly affecting the long-term health and well-being of rabbits. In this regard, dietary supplementation with CBD could be a useful tool to increase the resilience of animals in intensive farming conditions, where they are usually exposed to a wide range of stress factors.

### 4.4. Behavioral Analysis 

Behavioral analyses revealed significant changes in CBD-treated rabbits in terms of generalized increases in locomotor activity and grooming behaviors, which became more evident on day 9 than on day 1; indeed, on day 1, behaviors were relatively normal, while on day 9, data obtained indicate that CBD-treated rabbits showed significantly higher look around behavior than the CTRL group, suggesting an increased exploratory behavior. Although less evident, CBD-treated rabbits tended to show more pronounced “movement” behavior. A confirmation that CBD-treated rabbits spent significantly more time grooming than the CTRL group was given at the end of the experiment. The tendency towards more marked movement behavior, already observed on day 9, continued until the end of the experiment. “Stretching” also increased significantly in CBD-treated rabbits, which could be associated with changes in muscle tone or comfort. Such behavioral changes could be interpreted as an improvement in psychological well-being, probably associated with the anxiolytic action of CBD. Indeed, CBD has been successfully used to control anxiety and stressful conditions in animals [67,68], supporting the hypothesis that the behaviors observed in the rabbits of the present study could be signs of increased relaxation and comfort. Moreover, as shown in Figure 1, for the CTRL group, the level of movement remained relatively stable, fluctuating between 20 and 40 units over time. On the other hand, in the CBD group, there is a much more marked degree of variability, with an initial increase in movement to about 100 units at the second time point, with a decrease and then another increase to almost 100 units at the fourth time point. This suggests that CBD had a significant effect on the increase in movement of rabbits compared to the CTRL rabbits fed a diet without CBD, with variations occurring over time. The time spent lying down, showed a different trend, since the CTRL group remained approximately at the same level with slight fluctuations of about 7500–7800 units whilst the CBD-treated group showed a steady decline in time spent lying down, from an initial value almost equal to that of the CTRL rabbits and decreasing to about 5500 units at the fourth point. The variability in movement and the consistent reduction in lying time in the CBD group indicated that the addition of CBD in the diet may stimulate or activate physical activity over time, in stark contrast to the stability observed in the CTRL group. Further studies would be necessary to fully understand the mechanisms underlying these effects. 

### 4.5. Total Lipids and Fatty Acid Profile of the Loins, Thighs, and Livers 

Furthermore, it is also interesting to investigate the potential effect of CBD on lipid metabolism in rabbits and, consequently, on the lipid composition of the final products [27]. Starting from a wider context, the nutritional quality of rabbit meat can be further improved through targeted dietary strategies. Additives such as essential oils and plant extracts offer antimicrobial, antioxidant, anti-inflammatory, and immunomodulatory effects, while also serving as potential alternatives to antibiotics, contributing to livestock sustainability [6,10]. Thus, functional ingredients, including plant- and animal-derived bioactives, are increasingly used in feed and food applications for their ability to enhance the nutritional value, sensory attributes, and health benefits of animal products. Moreover, the administration of selected dietary additives in rabbits’ diet could be considered an effective way to enrich rabbit meat with unsaturated fatty acids, which are safe for human consumption [69]. This modification can intervene in different ways such as by free-ranging rabbits, which improves the concentration of n-3 PUFAs in the meat and the antioxidant capacity compared to cage rearing for the higher quality of nutrition based on grazed plants; or by the enrichment of the animals’ diets with natural feed [27], such as bilberry, thyme, and linseed, which improve the n-3 PUFA and antioxidant contents in the meat. For instance, Simonová et al. (2020) [70] noted that sage administration to rabbits increased the fat and protein contents, the meat energy value, and the eicosapentaenoic acid. Therefore, phytobiotics, plant-derived substances with health-promoting properties, such as hemp-derived CBD extracts are attracting growing interest in animal nutrition, and our study contributes to exploring their potential applications also in these terms.

Our results showed that the fatty acid profiles of the meat and liver samples reflected the diet, which was rich in n-6 PUFAs. The predominant fatty acids in the meat and liver were linoleic (C18:2n-6), oleic (C18:1n-9), palmitic (C16:0), and stearic (C18:0) acids. While n-3 PUFAs were present and represented mainly by C18:3n-3, they were less abundant, leading to a higher n-6/n-3 ratio in both cuts and liver. There is considerable evidence indicating that the fatty acids of dietary fats may significantly affect adipose and muscle fatty acid composition in rabbits [71]. The composition of the CBD extract, which was rich in linoleic acid but contained limited n-3 PUFAs, may have also contributed to the observed fatty acid profile. This little enhancement of n-3 PUFAs might be justified as the CBD extract used was a component of hemp oil rather than the oil itself, which usually contains high n-3 PUFA concentrations [72,73]. Otherwise, since the dietary effect was minimal, this can likely be attributed to the low level of CBD inclusion, maybe indicating the possibility of a dose-dependent relationship.

Nonetheless, the scarcity of n-3 PUFAs and the greater presence of n-6 PUFAs should not be considered only as a negative effect. Linoleic acid (LA, C18:2n-6) is an essential n-6 PUFA serving as a precursor to arachidonic acid (AA, C20:4n-6), which plays a key role in various physiological processes. According to Kim and Song (2024) [74], dietary LA and alpha-linolenic acid (ALA, C18:3n-3) are the primary n-6 and n-3 PUFAs, respectively, both critical for synthesizing long-chain PUFAs like AA, eicosapentaenoic acid (EPA), and docosahexaenoic acid (DHA). However, the conversion efficiency of these precursors into LC-PUFAs is low, making dietary sources important. As emphasized by Castellini et al. (2022) [75], humans and animals cannot synthesize LA or ALA due to the absence of necessary desaturase and elongase enzymes, so these fatty acids must be obtained from the diet. However, excessive intake of LA can hinder the conversion of ALA into EPA and DHA, as discussed by Kim and Song (2024) [75], and this is of particular concern in Western diets. Moreover, the intramuscular fat significantly influences meat quality, and alterations in its content can modify the fatty acid composition, affecting the nutritional quality of meat [76]. In our study, rabbit meat, rich in LA, especially in loins and thighs, serves as a functional food source for this essential PUFA. Jackson et al. (2024) [77] highlighted that elevated LA levels in the diet and blood are associated with improved cardiometabolic outcomes, and this is beneficial for health-conscious consumers concerned about excessive fat intake and its associated risks, such as ischemic heart disease, obesity, and type-2 diabetes [78].

Even though the differences were minor, the significantly higher level of C14:0 and C16:1n-7 in the CBD liver was associated with the dietary factor, while C10:0 and C12:0 levels in the loin were greater in the CTRL than in the CBD group. These findings may suggest that CBD treatment might have influenced the lipid composition, potentially modulating polyunsaturated fatty acid metabolism [79].

Regarding the effect of sex, a more pronounced influence was observed in the thigh, with significant differences recorded for some fatty acids (C17:0, C18:0, and C20:2n-6), while other fatty acids (C10:0, C12:0, and C16:1n-7) exhibited a trend toward significant variation. These differences may stem from distinct metabolic pathways or sex-based variations in fatty acid storage, as supported by previous research on gender-driven metabolic responses in rabbits [80,81].

Although most of our results did not reach statistical significance, it is still possible to formulate some hypotheses regarding the potential role of CBD in rabbit lipid metabolism through pathways related to oxidative stress modulation, endocannabinoid signaling, and inflammatory responses. Given the current lack of literature on this topic in rabbits, these interpretations remain speculative and should be viewed as preliminary considerations aimed at stimulating further research. Thus, the hypotheses here underwritten are intended as a contribution to ongoing scientific dialogue rather than as definitive conclusions.

So, several observations and existing evidence provide a basis for further exploration of cannabidiol’s (CBD) role in lipid metabolism, oxidative stress, and lipid oxidation. Lipid metabolism is fundamental in mammals, serving as the primary energy reserve and maintaining energy homeostasis. Key tissues involved include adipose tissue, skeletal muscle, and liver. In adipose tissue, fatty acids are mobilized to supply energy to other tissues. In skeletal muscle, fatty acids undergo oxidation, while in the liver, they contribute to the synthesis of triglycerides and the secretion of very low-density lipoproteins [80,82].

Peroxisome proliferator-activated receptors (PPARs), particularly PPAR-α and PPAR-γ, play a pivotal role in regulating lipid metabolism. PPAR-α, predominantly expressed in the liver, adipose tissue, and skeletal muscle, promotes fatty acid oxidation. Conversely, PPAR-γ, highly expressed in adipose tissue, regulates adipogenesis and lipid storage [80]. These transcription factors orchestrate the expression of genes responsible for fatty acid transport, storage, and oxidation, ensuring lipid homeostasis [82]. CBD has been shown to interact with various receptor targets, including PPAR-γ, CB2 receptors, and the 5-hydroxytryptamine 1A receptor (5-HT1A). Studies suggest that its effects on PPAR-γ may reduce lipid accumulation and improve metabolic parameters under certain conditions, such as obesity and hyperlipidemia [83]. Thus, it has been suggested that CBD may interact with PPAR-γ, thereby influencing lipid homeostasis, although direct evidence specific to rabbit models is currently lacking. Further research is needed to determine whether such interactions occur in this species and whether they significantly affect lipid deposition in tissues.

Similarly, the potential modulation of the endocannabinoid system (ECS) by CBD warrants attention. The ECS also plays a central role in regulating energy and lipid metabolism [84,85]. The ECS consists of endogenous cannabinoids like anandamide (AEA) and 2-arachidonoylglycerol (2-AG), their receptors (CB1 and CB2), and enzymes responsible for their synthesis and degradation [20]. AEA, derived from arachidonic acid, is critical in fat storage and energy balance. Although CBD exhibits low affinity for CB1 and CB2 receptors, it indirectly modulates the ECS by inhibiting fatty acid amide hydrolase (FAAH), increasing AEA levels. This interaction may influence lipid metabolism and energy expenditure [86]. 

Further connections between CBD and lipid homeostasis involve polyunsaturated fatty acid (PUFA) metabolism. Essential PUFAs, such as linoleic acid and α-linolenic acid, serve as precursors to n-6 and n-3 fatty acids, respectively. Metabolites of n-6 PUFAs, including AEA and 2-AG, contribute to inflammation and ECS tone. By modulating ECS activity and PUFA metabolism, cannabinoids like CBD may influence systemic inflammation, oxidative stress, and lipid balance [84]. However, the extent to which these interactions translate to observable changes in lipid profiles in rabbit tissues remains speculative and requires targeted studies.

Experimental evidence also highlights CBD’s effects on fatty acid metabolism. For example, Bielawiec et al. (2023) [86] demonstrated that CBD reduced the expression of long-chain fatty acid (LCFA) transport proteins and inhibited de novo lipogenesis in skeletal muscle. In rats fed a high-fat diet, CBD positively impacted fatty acid elongation and desaturation, reducing intramuscular lipid accumulation. Similarly, Jadoon et al. (2016) [87] observed that CBD (dosage of 3 mg/kg body weight/day over a period of 4 weeks) improved lipid profiles, including elevated HDL-C levels and reduced total cholesterol and liver triglycerides, in genetically obese mice.

The role of CBD in cholesterol metabolism has also been explored. He et al. (2024) [88] reported that CBD enhances cholesterol efflux through the PPAR-γ-ABCA1/ABCG1 pathway, crucial for macrophage cholesterol regulation. Additionally, CBD downregulated scavenger receptors such as CD36 and SRA while upregulating cholesterol efflux proteins like ABCA1 and ABCG1, reducing foam cell formation and intracellular cholesterol accumulation. These mechanisms highlight CBD’s potential in improving cardiovascular health and mitigating atherosclerosis.

While these mechanisms have been observed in vitro and in other animal models, their relevance to rabbit tissues and their potential implications for meat quality remain unexplored.

However, challenges related to the oral administration of CBD in rabbits remain significant. Rooney et al. (2022) [30] noted that rabbits exhibit low bioavailability of CBD and its precursor CBDA due to their unique coprophagic behavior, which likely alters the absorption profiles. While dietary lipids have been shown to enhance CBD absorption in rats, similar strategies require adaptation for rabbit models. This variability in pharmacokinetics may explain the lack of significant outcomes in our study, underscoring the need for tailored administration strategies.

### 4.6. Lipid Oxidative Status of the Muscles and Livers

Finally, according to Wang et al. (2021) [89], rabbit meat (rich in PUFAs) is prone to lipid oxidation. In our results, the liver of the CBD-fed rabbits showed less conjugated dienes than the other group. This might be linked to the fact that oxidative stress, a critical factor in cellular homeostasis, is another area where CBD exhibits potential effects. Excessive Reactive Oxygen Species (ROS) production disrupts redox balance, leading to lipid peroxidation, DNA damage, and inflammation. Phyto cannabinoids like CBD mitigate oxidative stress through multiple mechanisms, including ROS scavenging, inhibition of NADPH oxidase activity, and modulation of ECS signaling [90]. Additionally, endocannabinoids, like AEA (anandamide or arachidonoyl ethanolamine), attenuate the oxidative damage, suggesting a complex interplay between ECS and oxidative pathways. Whether these effects are sufficiently pronounced to alter the oxidative stress markers in rabbit tissues is yet to be determined. 

Moreover, studies by Schuelert and McDougall (2011) [91] have suggested that CBD exerts significant antioxidant properties, which could be useful for mitigating oxidative stress. The decrease in CD level observed in this trial suggests that CBD exhibited some antioxidant activity, which may be helpful in avoiding oxidative damage [92]. However, further investigation is needed to explain the mechanisms by which these changes in fatty acid composition could be influenced by CBD and to find out whether these trends turn into significant benefits in rabbit health, welfare, and meat properties. 

## 5. Conclusions

This study provides new insights into the effects of CBD supplementation in rabbits, highlighting its potential role in improving animal health and welfare. CBD was associated with positive physiological and behavioral responses, improved body weight gain, reduced triglyceride levels, and enhanced antioxidant capacity, as shown by lower dROM and higher BAP values. Importantly, CBD inclusion did not negatively affect the lipid quality or chemical composition of rabbit meat. These findings suggest that CBD may act as a modulator of lipid metabolism and oxidative stress, offering a promising alternative to traditional feed additives for intensive rabbit farming, where animals are often exposed to stress factors. However, more detailed studies are required to understand the role of CBD in rabbit lipid metabolism and to assess whether it can effectively improve the nutritional and functional properties of the meat itself, with possible implications for key aspects such as shelf life, food safety, and consumer perception. Furthermore, future studies should aim to optimize CBD administration strategies, further investigate its bioavailability and interactions with the endocannabinoid system, and assess its long-term effects on productivity and welfare. Additionally, the gender-specific differences observed in some parameters merit further investigation. 

## Figures and Tables

**Table 1:** CBD extract chemical analysis.

Total cannabidiol (CBD + CBA)	10.35%
Cannabidiol, CBD	9.42%
Cannabidiolic acid, CBA	1.06%
Total tetrahydrocannabinol (THC + THCA)	0.19%
D9-Tetrahydrocannabinol	0.19%
Tetrahydrocannabinolic acid	ND
D8-Tetrahydrocannabidinol	ND
Total cannabigerol (CBG + CBGA)	0.36%
Cannabigerol	0.24%
Cannabierolic acid	0.11%
Cannabinol	0.04%
Cannabichromene	0.03%
Cannabidivarin	0.07%
Cannabidivarinic acid	ND

Abbreviations: ND = not detected.

**Table 2:** Chemical-nutritional characteristics and ingredients of the basal diet and wafer used during the trial.

	Basal Diet ^1^	Wafer ^2^
Crude protein, % AF	15.6	11.8
Ether extract, % AF	3.2	1.5
Crude fiber, % AF	19.9	19.8
Ashes, % AF	6.6	8.4
Ca, % AF	0.34	-
P, % AF	0.46	0.13
Na, % AF	0.22	0.05
Lysine, % AF	0.66	-
Methionine, % AF	0.23	-
DE, MJ/kg	11.6	10.9

Abbreviations: AF = as feed; DE = Digestible energy. ^1^ Ingredients (in a decreasing-amount order): wheat bran, sunflower meal, dehydrated beet pulp, wheat straw, soybean shells, barley, grape seeds meal, toasted soybean seeds, sugar cane molasses, dehydrated alfalfa meal, soybean oil, salt. Min-vit supplementation/kg: Vit A 9000 IU, Vit D3 900 IU, Vit E 30 mg, Mn 30 mg, Zn 42 mg, Cu 6 mg, Fe 45 mg, I 1.8 mg, Se 0.06 mg. ^2^ Ingredients (in a decreasing-amount order): dehydrated alfalfa hay, dehydrated alfalfa, heat-crushed barley, wheat bran, oats, corn flakes, sugar cane molasses, glucose and fructose syrup, maltodextrins, sucrose. Min-vit supplementation/kg: Vit A 367 IU, Vit D3 37.65 IU, choline 2.21 mg, Vit B1 0.61 mg, Vit B2 0.59 mg, Vit E 9.80 mg, biotin 0.02 mg, folic acid 0.04 mg, niacin 2.45 mg, calcium D-pantotenate 0.04 mg, CuSO_4_ 0.37 mg, Fe 0.49 mg, MnO 2.54 mg, ZnSO_4_ * H_2_O 2.13 mg, butylhydroxitolouene 3.00 mg.

**Table 3:** Fatty acid profile of the CBD extract (g fatty acids/100 g total FAME).

Fatty Acid	Mean Value
C6:0	0.03
C8:0	41.83
C10:0	39.78
C12:0	0.15
C14:0	0.01
C16:0 iso	0.04
C16:0	1.20
C16:1n-7	0.01
C18:0	0.44
C18:1n-9	1.57
C18:1n-7	0.15
C18:2n-6	9.76
C18:3n-6	0.85
C18:3-n3	3.58
C18:4n-3	0.28
C20:0	0.13
C20:1n-9	0.05
C22:0	0.05
C22:5n-3	0.06
SFA	83.67
MUFA	1.79
n-6 PUFA	10.62
n-3 PUFA	3.92

SFA: saturated fatty acids, MUFA: monounsaturated fatty acids, PUFA: polyunsaturated fatty acids.

**Table 4:** Fatty acid profile (g fatty acids/100 g total fatty acid methyl esters, FAME) of the diet.

Fatty Acid	Mean Value
C10:0	0.16
C12:0	0.18
C14:0	0.53
C14:1n-5	0.02
iso-C15:0	0.05
anteiso-C15:0	0.02
C15:0	0.14
iso-C16:0	0.01
C16:0	19.53
C16:1n-9	0.14
C16:1n-7	0.42
C17:0	0.15
C17:1	0.01
C18:0	3.90
C18:1n-9	26.71
C18:1n-7	1.17
C18:2n-6	39.84
C18:3n-6	0.10
C18:3n-3	4.89
C20:0	0.34
C20:1n-11	0.03
C20:1n-9	0.38
C20:1n-7	0.01
C20:2n-6	0.05
C20:3n-6	0.02
C20:4n-6	0.21
C22:0	0.32
C22:1n-11	0.10
C22:1n-9	0.23
C22:4n-6	0.05
C22:5n-3	0.03
C24:0	0.27
SFA	25.61
MUFA	29.22
n-6 PUFA	40.26
n-3 PUFA	4.91

SFA: saturated fatty acids, MUFA: monounsaturated fatty acids, PUFA: polyunsaturated fatty acids.

**Table 5:** Rabbit in vivo performance according to dietary treatment group, sex, and their interaction.

	Group		Sex		*p*-Values			RMSE
	CTRL	CBD	Females	Males	Group	Sex	Interaction	
LW 65d, g	1727.1	1707.1	1625.7 ^b^	1808.6 ^a^	0.95	0.03	0.38	91.6
LW 92d, g	2637.1 ^b^	2731.4 ^a^	2608.6 ^b^	2760.0 ^a^	0.05	0.03	0.62	101.3
BWG 65–70d, g	35.71 ^b^	48.27 ^a^	40.29 ^b^	43.71 ^a^	0.01	0.04	0.49	3.96
BWG 86–92d, g/d	38.98 ^B^	44.08 ^A^	46.33 ^A^	36.74 ^B^	<0.01	<0.01	0.51	3.26
BWG 65–92d, g/d	33.70 ^B^	37.94 ^A^	36.40	35.23	<0.01	0.12	0.28	2.75
FI 65–70d, g/d	126.6 ^B^	145.4 ^A^	135.1	136.9	<0.01	0.63	0.38	10.0
FI 71–78d, g/d	153.7	153.9	145.3 ^B^	162.3 ^A^	0.54	<0.01	0.79	11.3
FI 79–85d, g/d	170.7 ^A^	157.1 ^B^	147.6 ^B^	180.2 ^A^	<0.01	<0.01	0.61	13.5
FI 86–92d, g/d	186.8	179.2	169.3 ^B^	196.8 ^A^	0.11	<0.01	0.74	12.63
FI 65–92d, g/d	161.9	161.4	151.8 ^B^	171.5 ^A^	0.45	<0.01	0.57	11.9
FCR 65–70d, g/g	3.54 ^A^	3.02 ^B^	3.35	3.13	<0.01	0.07	0.70	0.26
FCR 71–78d, g/g	4.30	4.14	4.13	4.30	0.15	0.012	0.80	0.32
FCR 79–85d, g/g	5.51	5.27	5.01 ^B^	5.77 ^A^	0.06	<0.01	0.47	0.39
FCR 86–92d, g/g	4.79 ^A^	4.06 ^B^	3.65 ^B^	5.35 ^A^	<0.01	<0.01	0.82	0.30
FCR 65–92d, g/g	4.80 ^A^	4.25 ^B^	4.17 ^B^	4.86 ^A^	<0.01	<0.01	0.63	0.32

CBD: cannabidiol extract; RMSE: root mean square error; ^a, b^: *p* < 0.05, ^A, B^: *p* < 0.01; LW: live weight; BWG: body weight gain; FI: feed intake; FCR: feed conversion ratio.

**Table 6:** Rabbit carcass traits according to dietary treatment group, sex, and their interaction.

	Group		Sex		*p*-Values			RMSE
	CTRL	CBD	Females	Males	Group	Sex	Interaction	
LW, g	2617.1	2668.3	2585.2 ^b^	2700.2 ^a^	0.06	0.03	0.79	165.4
Skin, % LW	14.23	15.31	13.72	15.81	0.19	0.06	0.27	1.05
Full GUT, % LW	17.65	16.69	17.86 ^a^	16.49 ^b^	0.72	0.04	0.36	1.39
Empty GUT, % LW	8.80	8.44	8.76	8.49	0.42	0.51	0.24	0.87
Warm Carcass, g	1604.3 ^a^	1702.9 ^b^	1581.4 ^a^	1725.8 ^b^	0.03	0.02	0.75	109.1
Carcass Yield, %	61.30 ^B^	63.82 ^A^	61.17 ^B^	63.91 ^A^	<0.01	<0.01	0.16	1.65
Spleen, % HC	4.25 ^B^	6.70 ^A^	5.41	5.54	<0.01	0.59	0.20	0.23
Urinary + genitals, % LW	0.66	0.62	0.64	0.63	0.74	0.95	0.49	0.04
pH LD1h	7.11	6.86	6.97	7.06	0.11	0.80	0.95	0.33
pH BF1h	6.91	6.75	6.71	6.96	0.56	0.30	0.44	0.40
Chilled carcass, g	1577.1	1688.6	1591.4	1674.3	0.07	0.08	0.91	98.6
CC Yield, %	60.26 ^B^	63.28 ^A^	61.56	62.00	<0.01	0.63	0.84	0.47
pH LD24h	5.87 ^A^	5.76 ^B^	5.81	5.83	<0.01	0.94	0.31	0.045
pH BF24h	5.93	5.89	5.91	5.92	0.28	0.95	0.43	0.048
Reference carcass, g	1029.6 ^b^	1116.5 ^a^	1022.1 ^b^	1124.0 ^a^	0.03	0.03	0.70	84.93
Head, % RC	14.33	13.04	14.02	14.15	0.24	0.63	0.35	1.06
Liver, % RC	8.47 ^A^	6.91 ^B^	7.55	7.82	<0.01	0.92	0.20	0.43
Kidney, % RC	1.71	1.60	1.66	1.66	0.22	0.85	0.46	0.14
Lungs, % RC	1.43	1.43	1.41	1.45	0.96	0.66	0.59	0.12
Heart, % RC	1.29	1.33	1.26	1.36	0.31	0.28	0.23	0.09
Inguinal fat % RC	0.19 ^A^	0.15 ^B^	0.18	0.16	0.01	0.45	0.84	0.01
Perirenal fat, % RC	2.39	2.40	2.55 ^a^	2.23 ^b^	0.96	0.02	0.76	0.16
Scapular fat, % RC	0.49	0.49	0.50	0.47	0.97	0.80	0.94	0.03

CBD: cannabidiol extract; RMSE: root mean square error; ^a, b^: *p* < 0.05, ^A, B^: *p* < 0.01; LW: live weight; GUT: gastro-intestinal tract; CC: chilled carcass; RC: reference carcass; HC: hot carcass; LD: *Longissimus dorsi*; BF: *Biceps femoris*.

**Table 7:** Seconds spent by rabbits in different behaviors according to dietary treatment group, sex and their interaction at day 1 of the trial.

	Group		Sex		*p*-Values			RMSE
	CTRL	CBD	Female	Male	Group	Sex	Interaction	
Grooming	974.0	1064.5	1131.8	906.2	0.8024	0.3719	0.5942	482.9
Moving	35.74	36.23	29.73	42.27	0.8330	0.0706	0.0306	11.66
Setting	610.7	816.1	740.2	686.6	0.2162	0.6184	0.7890	305.03
Lying	7534.6	7850.8	7393.8	7891.7	0.4336	0.3243	0.8155	799.4
Look around	4.27	5.79	6.24	3.84	0.4786	0.1932	0.1917	2.99
Gnawing	3.90	3.57	4.74	2.73	0.9890	0.5115	0.9167	5.49
Stretching	28.99	24.03	23.44	29.57	0.4394	0.4241	0.7208	15.51
Sniffing	121.6	120.9	129.4	112.2	0.9574	0.3360	0.9717	31.86

CBD: cannabidiol extract group; RMSE: root mean square error.

**Table 8:** Seconds spent by rabbits in different behaviors according to dietary treatment group, sex, and their interaction at day 9 of the trial.

	Group		Sex		*p*-Values			RMSE
	CTRL	CBD	Female	Male	Group	Sex	Interaction	
Grooming	1074.6	1155.8	1244.3	986.1	0.7375	0.3150	0.5688	440.67
Moving	22.29 ^b^	90.09 ^a^	39.91	72.46	0.0192	0.3440	0.2839	10.31
Setting	784.5	564.6	698.8	650.4	0.1640	0.6088	0.9097	285.4
Lying	7689.1	7267.1	7212.5	7743.7	0.4552	0.3154	0.8271	842.3
Look around	0.46 ^B^	11.80 ^A^	10.86 ^a^	1.40 ^b^	0.0011	0.0450	0.4079	2.86
Gnawing	1.51	4.43	4.53	1.40	0.4817	0.4378	0.9361	6.38
Stretching	24.97	35.31	30.49	29.80	0.2846	0.9321	0.5799	17.13
Sniffing	115.5	187.8	163.97	139.29	0.1487	0.7506	0.8829	83.05

CBD: cannabidiol extract group; RMSE: root mean square error; ^a, b^: *p* < 0.05, ^A, B^: *p* < 0.01.

**Table 9:** Seconds spent by rabbits in different behaviors according to dietary treatment group, gender and their interaction at end of the trial.

	Group		Sex		*p*-Values			RMSE
	CTRL	CBD	Female	Male	Group	Sex	Interaction	
Grooming	1019.2 ^b^	1820.5 ^a^	1635.5	1204.1	0.0498	0.3619	0.9746	626.95
Moving	33.40	101.03	54.23	80.14	0.0927	0.3759	0.4383	72.58
Setting	923.7	967.9	946.8	944.7	0.8791	0.9884	0.6739	531.5
Lying	7458.2	5875.6	6340.3	6993.5	0.0850	0.5956	0.8224	1472.8
Look around	11.20	37.06	24.11	24.14	0.0772	0.7825	0.8891	24.82
Gnawing	6.11	14.03	13.91	6.23	0.5397	0.5559	0.7101	20.29
Stretching	27.31 ^b^	44.11 ^a^	38.71	32.74	0.0101	0.4915	0.2946	9.58
Sniffing	94.0	107.6	82.91	118.7	0.6736	0.4037	0.0928	81.78

CBD: cannabidiol extract group; RMSE: root mean square error; ^a, b^: *p* < 0.05.

**Table 10:** Rabbit blood profiles according to dietary treatment group, sex, and their interaction.

	Group		Sex		*p*-Values			RMSE
	CTRL	CBD	Female	Male	Group	Sex	Interaction	
Glu, mg/dL	107.9	97.34	99.30	105.9	0.1381	0.4062	0.1219	11.19
Chol, mg/dL	66.94	57.88	64.64	59.19	0.0853	0.1689	0.5971	9.87
Try, mg/dL	82.50 ^A^	72.33 ^B^	78.63	76.20	0.0217	0.3398	0.8062	7.32
TP, g/L	7.20	7.29	7.29	7.19	0.8676	0.8077	0.0735	0.70
Alb, g/L	3.00	2.87	2.84	3.03	0.5011	0.3042	0.3546	0.29
Glob, g/d	4.20	4.41	4.45	4.16	0.5946	0.4235	0.0163	0.5853
Alb/Glob	0.71 ^a^	0.65 ^b^	0.64 ^b^	0.73 ^a^	0.0221	0.0296	0.7859	0.059
Urea, mg/dL	30.43	29.14	31.14	28.43	0.6801	0.4791	0.6089	7.45
Crea, mg/dL	0.877	0.867	0.94	0.81	0.8217	0.3088	0.3684	0.24
Ast, U/L	25.16	28.14	28.54	24.76	0.5301	0.3926	0.0408	7.10
Alt, U/L	65.36	61.73	63.06	64.03	0.2101	0.8654	0.3005	4.92
Ldh, U/L	460.29	468.57	437.0	491.86	0.7235	0.2346	0.0148	83.76
CPK, U/L	1260.7	1324.3	1312.7	1272.3	0.6247	0.7898	0.0857	216.44
dROMS UCARR	103.7 ^A^	69.71 ^B^	88.71	84.74	0.0022	0.3215	0.5430	16.05
BAP, umol/L	1636.7 ^B^	2543.3 ^A^	2241.9	1938.1	0.0030	0.4503	0.9940	419.18

Glu: glucose, Chol: cholesterol, Try: triglycerides, TP total proteins, Alb: albumin, Glob: globulin, Crea: creatinine, Ast: aspartate amino transferase, Alt: alanine-aminotransferase, Ldh: lactate dehydrogenase, CPK: creatine phosphokinase, dROMS: Reactive Oxygen Metabolites-derived compounds, BAP: Biological Antioxidant Potential. CBD: cannabidiol extract group; RMSE: root mean square error; ^a, b^: *p* < 0.05, ^A, B^: *p* < 0.01.

**Table 11:** Significant identified metabolites downregulated (↓) by CBD vs. CTRL in rabbits. Statistical significance was tested by *t*-test (*p* < 0.05; fold change > 0.2).

Compound	Regulation CBD vs. CTRL
D-(-)-Fructofuranose 5TMS (isomer 1)	↓
Dihydrocholesterol, TMS	↓
2-Monolinoleoylglycerol, 2TMS	↓

**Table 12:** Total lipid content (g/100 g tissue) and fatty acid profile (g fatty acid/100 g FAME) of the loins according to dietary treatment group, sex, and their interaction.

	Group		Sex		*p* Values			RMSE
	CTRL	CBD	Female	Male	Group	Sex	Interaction	
**Total lipids**	2.13	1.26	1.52	1.87	0.135	0.529	0.694	1.004
**Fatty acids**								
C10:0	0.12 ^a^	0.05 ^b^	0.07	0.10	0.024	0.205	0.069	0.047
C12:0	0.17 ^a^	0.09 ^b^	0.11	0.15	0.019	0.198	0.119	0.052
Iso C14:0	0.03	0.03	0.03	0.03	0.675	0.224	0.115	0.011
C14:0	1.61	1.55	1.48	1.68	0.846	0.478	0.896	0.494
C14:1n-5	0.05	0.04	0.04	0.05	0.535	0.421	0.599	0.03
Iso C15:0	0.05	0.05	0.04	0.05	0.806	0.591	0.527	0.015
Anteiso C15:0	0.12	0.12	0.12	0.12	0.933	0.677	0.227	0.018
C15:0	0.58	0.57	0.59	0.57	0.819	0.421	0.844	0.046
Iso C16:0	0.16	0.15	0.16	0.15	0.568	0.384	0.754	0.029
C16:0	23.81	24.60	24.07	24.35	0.273	0.689	0.272	1.257
C16:1n-9	0.38	0.36	0.37	0.37	0.372	0.919	0.919	0.044
C16:1n-7	1.37	1.36	1.12	1.60	0.978	0.147	0.828	0.564
C17:0	0.65	0.66	0.70 ^a^	0.61 ^b^	0.851	0.033	0.620	0.068
C18:0	7.77	8.35	8.61	7.51	0.415	0.140	0.829	1.269
C18:1n-9	23.87	23.43	23.28	24.01	0.720	0.553	0.579	2.215
C18:1n-7	1.46	1.53	1.48	1.50	0.599	0.874	0.642	0.219
C18:2n-6	27.97	27.29	27.48	27.78	0.444	0.729	0.221	1.568
C18:3n-6	0.08	0.11	0.11	0.08	0.243	0.243	0.929	0.034
C18:3n-3	2.45	2.35	2.30	2.49	0.806	0.644	0.544	0.743
C20:0	0.14	0.14	0.15	0.12	0.925	0.130	0.887	0.032
C20:1n-9	0.22	0.23	0.22	0.23	0.724	0.442	0.506	0.042
C20:2n-6	0.19	0.19	0.17 ^b^	0.21 ^a^	0.624	0.015	0.099	0.031
C20:3n-6	0.32	0.37	0.34	0.35	0.577	0.852	0.510	0.145
C20:4n-6	4.47	4.43	4.86	4.04	0.974	0.590	0.635	2.723
C20:3n-3	0.05	0.04	0.04	0.05	0.375	0.375	0.283	0.015
C20:4n-3	0.01	0.01	0.01	0.01	0.717	0.717	0.124	0.008
C20:5n-3	0.09	0.10	0.10	0.09	0.692	0.692	0.494	0.051
C22:0	0.05	0.06	0.05	0.05	0.338	0.766	0.898	0.017
C22:1n-9	0.05	0.05	0.05	0.04	0.716	0.347	0.102	0.023
C22:4n-6	0.80	0.82	0.84	0.78	0.934	0.799	0.577	0.455
C22:5n-6	0.27	0.27	0.29	0.25	0.913	0.631	0.775	0.144
C22:5n-3	0.44	0.42	0.47	0.40	0.864	0.563	0.578	0.263
C24:0	0.11	0.12	0.12	0.10	0.747	0.665	0.088	0.054
C22:6n-3	0.09	0.04	0.09	0.04	0.223	0.223	0.815	0.064
C24:1n-9	0.03	0.04	0.04	0.03	0.820	0.460	0.820	0.023
SFA	35.34	36.53	36.30	35.58	0.085	0.275	0.177	1.156
MUFA	27.43	27.03	26.61	27.85	0.778	0.391	0.651	2.567
n-6 PUFA	34.10	33.48	34.08	33.50	0.711	0.732	0.937	3.027
n-3 PUFA	3.12	2.96	3.01	3.07	0.493	0.807	0.571	0.428
n-6 PUFA + n-3 PUFA	37.22	36.40	37.10	36.60	0.620	0.796	0.865	2.858
n-6/n-3	11.25	11.40	11.6	11.1	0.906	0.713	0.717	2.281

SFA: saturated fatty acids, MUFA: monounsaturated fatty acids, PUFA: polyunsaturated fatty acids. CBD: cannabidiol extract group; RMSE: root mean square error; ^a, b^: *p* < 0.05.

**Table 13:** Total lipid content (g/100 g tissue) and fatty acid profile (g fatty acid/100 g fatty acid methyl esters) of the thighs according to dietary treatment group, sex, and their interaction.

	Group		Sex		*p* Values			RMSE
	CTRL	CBD	Female	Male	Group	Sex	Interaction	
**Total lipids**	1.56	1.62	1.54	1.64	0.725	0.604	0.867	0.331
**Fatty acids**								
C10:0	0.11	0.07	0.06	0.12	0.114	0.060	0.067	0.048
C12:0	0.16	0.11	0.10	0.16	0.105	0.087	0.152	0.054
Iso C14:0	0.02	0.03	0.02	0.02	0.231	0.574	0.910	0.007
C14:0	1.39	1.48	1.30	1.58	0.674	0.218	0.823	0.389
C14:1n-5	0.07	0.05	0.05	0.08	0.368	0.239	0.516	0.040
Iso C15:0	0.05	0.05	0.05	0.05	0.444	0.912	0.740	0.014
Anteiso C15:0	0.12	0.11	0.12	0.11	0.760	0.760	0.544	0.019
C15:0	0.55	0.56	0.56	0.55	0.769	0.560	0.826	0.041
Iso C16:0	0.14	0.14	0.15	0.14	0.884	0.470	0.942	0.021
C16:0	23.34	23.95	23.40	23.90	0.388	0.480	0.315	1.259
C16:1n-9	0.39	0.38	0.38	0.39	0.728	0.944	0.351	0.065
C16:1n-7	1.63	1.44	1.23	1.84	0.581	0.089	0.428	0.602
C17:0	0.63	0.65	0.69 ^a^	0.59 ^b^	0.522	0.017	0.522	0.062
C18:0	8.63	8.67	9.24 ^a^	8.06 ^b^	0.937	0.042	0.663	0.930
C18:1n-9	20.81	21.67	20.37	22.11	0.525	0.215	0.639	2.420
C18:1n-7	1.55	1.47	1.51	1.51	0.296	0.950	0.886	0.131
C18:2n-6	28.53	28.90	29.91	28.52	0.628	0.601	0.437	1.357
C18:3n-6	0.10	0.10	0.11	0.09	0.803	0.292	0.427	0.027
C18:3n-3	2.16	2.33	2.14	2.35	0.525	0.406	0.702	0.465
C20:0	0.13	0.13	0.14	0.12	0.904	0.094	0.500	0.019
C20:1n-9	0.20	0.22	0.19	0.23	0.387	0.074	0.348	0.038
C20:2n-6	0.24	0.26	0.21 ^B^	0.28 ^A^	0.376	0.006	0.150	0.037
C20:3n-6	0.54	0.44	0.50	0.48	0.191	0.830	0.919	0.133
C20:4n-6	5.80	4.62	5.91	4.50	0.319	0.232	0.739	2.056
C20:3n-3	0.05	0.06	0.05	0.06	0.616	0.292	0.973	0.022
C20:4n-3	0.01	0.01	0.01	0.01	0.467	0.467	0.467	0.008
C20:5n-3	0.10	0.09	0.10	0.08	0.359	0.434	0.842	0.031
C22:0	0.06	0.05	0.06	0.05	0.579	0.116	0.278	0.016
C22:1n-9	0.04 ^a^	0.03 ^b^	0.04	0.03	0.048	0.330	0.842	0.008
C22:4n-6	1.10	0.88	1.05	0.93	0.285	0.543	0.671	0.361
C22:5n-6	0.42	0.31	0.40	0.34	0.324	0.113	0.093	0.821
C22:5n-3	0.64	0.50	0.67	0.47	0.365	0.225	0.450	0.284
C24:0	0.12	0.10	0.10	0.12	0.270	0.182	0.674	0.023
C22:6n-3	0.11	0.09	0.12	0.08	0.390	0.200	0.182	0.049
C24:1n-9	0.04	0.04	0.05	0.03	0.939	0.201	0.285	0.029
SFA	35.46	36.12	36.00	35.58	0.307	0.509	0.152	1.135
MUFA	24.73	25.30	23.82	26.21	0.719	0.154	0.562	2.867
n-6 PUFA	36.74	35.50	37.10	35.14	0.492	0.283	0.961	3.194
n-3 PUFA	3.08	3.08	3.08	3.07	0.996	0.914	0.667	0.265
n-6 PUFA + n-3 PUFA	39.80	38.60	40.20	38.20	0.489	0.315	0.989	3.179
n-6/n-3	12.04	11.58	12.14	11.48	0.581	0.433	0.746	1.496

SFA: saturated fatty acids, MUFA: monounsaturated fatty acids, PUFA: polyunsaturated fatty acids. CBD: cannabidiol extract group; RMSE: root mean square error; ^A, B^: *p* > 0.01; ^a, b^: *p* < 0.05.

**Table 14:** Total lipid content (g/100 g tissue) and fatty acid profile (g fatty acid/100 g fatty acid methyl esters) of the livers according to dietary treatment group, sex, and their interaction.

	Group		Sex		*p* Values			RMSE
	CTRL	CBD	Female	Male	Group	Sex	Interaction	
**Total lipids**	2.95	3.56	3.27	3.24	0.951	0.104	0.602	0.625
**Fatty acids**								
C10:0	0.02	0.01	0.02	0.01	0.256	0.260	0.563	0.005
C12:0	0.02	0.02	0.02	0.02	0.592	0.592	0.128	0.004
Iso C14:0	0.01	0.01	0.01	0.01	0.869	0.017	0.159	0.005
C14:0	0.31	0.34	0.36	0.30	0.319	0.563	0.737	0.110
C14:1n-5	0.01	0.01	0.01	0.01	0.070	0.778	0.113	0.005
Iso C15:0	0.02	0.02	0.02	0.02	0.588	0.588	0.473	0.008
anteiso C15:0	0.04	0.04	0.04	0.04	0.806	0.591	0.591	0.015
C15:0	0.38	0.38	0.40	0.36	0.156	0.868	0.659	0.054
Iso C16:0	0.08	0.07	0.09	0.07	0.123	0.722	0.830	0.021
C16:0	17.72	19.16	18.13	18.74	0.394	0.061	0.556	1.268
C16:1n-9	0.26	0.28	0.29	0.25	0.223	0.554	0.703	0.063
C16:1n-7	0.34	0.49	0.415 ^b^	0.418 ^a^	0.962	0.050	0.962	0.128
C17:0	1.03	1.01	1.05	0.99	0.260	0.809	0.983	0.103
C18:0	20.87	19.96	20.49	20.35	0.826	0.174	0.834	1.155
C18:1n-9	10.85	12.20	12.12	10.83	0.316	0.260	0.828	2.083
C18:1n-7	1.05	1.07	1.08	1.04	0.704	0.735	0.312	0.144
C18:2n-6	31.36	30.94	30.58	31.72	0.057	0.445	0.377	0.979
C18:3n-6	0.14	0.12	0.14	0.12	0.352	0.534	0.605	0.042
C18:3n-3	1.18	1.25	1.28	1.15	0.249	0.539	0.635	0.189
C20:0	0.13	0.13	0.14	0.13	0.475	0.743	0.050	0.011
C20:1n-9	0.18	0.18	0.16	0.20	0.339	0.895	0.529	0.057
C20:2n-6	0.57 ^a^	0.53 ^b^	0.41	0.68	0.037	0.727	0.231	0.211
C20:3n-6	0.86	0.76	0.72	0.90	0.220	0.514	0.584	0.263
C20:4n-6	9.56	8.46	9.25	8.78	0.609	0.244	0.780	1.647
C20:3n-3	0.06	0.04	0.05	0.06	0.310	0.105	0.645	0.026
C20:4n-3	0.01	0.02	0.02	0.01	0.829	0.293	1.000	0.007
C20:5n-3	0.06	0.06	0.07	0.06	0.339	0.889	0.385	0.016
C22:0	0.20	0.17	0.18	0.19	0.358	0.095	0.851	0.028
C22:1n-9	0.03	0.04	0.04	0.04	0.813	0.120	0.212	0.013
C22:4n-6	0.99	0.85	0.89	0.95	0.644	0.256	0.543	0.220
C22:5n-6	0.59	0.51	0.55	0.54	0.894	0.362	0.556	0.147
C22:5n-3	0.42	0.34	0.40	0.36	0.384	0.149	1.000	0.085
C24:0	0.19	0.17	0.19	0.18	0.715	0.519	0.692	0.005
C22:6n-3	0.21	0.16	0.21	0.15	0.067	0.117	0.697	0.054
C24:1n-9	0.26	0.18	0.21	0.23	0.808	0.151	0.339	0.090
SFA	41.02	41.51	41.13	41.40	0.452	0.608	0.730	1.176
MUFA	12.97	14.45	14.31	13.11	0.254	0.349	0.767	2.267
n-6 PUFA	44.07	42.17	42.54	43.69	0.134	0.344	0.642	2.156
n-3 PUFA	1.94	1.86	2.01	1.79	0.481	0.07	0.743	0.199
n-6 PUFA + n-3 PUFA	46.00	44.00	44.6	45.5	0.132	0.332	0.632	2.233
n-3/n-6	0.04	0.04	0.047 ^a^	0.041 ^b^	0.023	0.953	0.993	0.004
n-6/n-3	22.87	23.04	21.17 ^b^	24.74 ^a^	0.025	0.903	0.924	2.500

SFA: saturated fatty acids, MUFA: monounsaturated fatty acids, PUFA: polyunsaturated fatty acids. CBD: cannabidiol extract group; RMSE: root mean square error; ^a, b^: *p* < 0.05.

**Table 15:** Cholesterol content (mg/100 g tissue) of the loins, thighs and livers of the rabbits according to dietary treatment group, sex, and their interaction.

Meat Portion/Organ	Group		Sex		*p*-Values			RMSE
	CTRL	CBD	Female	Male	Group	Sex	Interaction	
Loin	41.253	36.238	37.049	40.442	0.240	0.418	0.077	7.436
Thigh	48.153	50.925	50.046	49.032	0.301	0.698	0.770	4.709
Liver	352.72	416.872	388.902	380.691	0.205	0.865	0.639	87.642

CBD: cannabidiol extract group; RMSE: root mean square error.

## Data Availability

The data presented in this study are available on request from the corresponding author.
