# Correction: Addeo et al. Dietary Cannabidiol Supplementation on Growth Performance, Behavior, Blood Profile, Metabolomic Analysis, and Fatty Acid Composition in Rabbits: A Multi-Disciplinary Approach to Improve Welfare and Productivity. *Vet. Sci.* 2025, *12*, 759

**DOI:** 10.3390/vetsci12090828

**Published:** 2025-08-28

**Authors:** Nicola Francesco Addeo, Valeria Iervolino, Ruggero Amato, Mariarosaria Lanzieri, Daria Lotito, Maria Vittoria Tignani, Alessia Staropoli, Sara Damiano, Pietro Lombardi, Francesco Vinale, Giuliana Parisi, Fulvia Bovera, Nadia Musco, Vincenzo Mastellone

**Affiliations:** 1 Department of Veterinary Medicine and Animal Production, University of Naples Federico II, 80137 Naples, Italy; 2 Department of Agriculture, Food, Environment and Forestry (DAGRI), University of Florence, Via delle Cascine 5, 50144 Florence, Italy

In the originally published version of this article [1], an earlier version of the manuscript was inadvertently used during the production process. As a result, several errors appeared in the published article that do not reflect the authors’ final, approved version.

Specifically, the following sections have been corrected:

Introduction: Updated to reflect the final approved text.

Materials and Methods: Minor textual revisions and clarifications incorporated.

Results and Discussion: Descriptions corrected to match the final version.

Conclusions: Minor textual revisions and clarifications incorporated.

Tables: 

Tables 2, 6 and 12–15 were replaced with the correct versions.

Tables 7–10 were replaced with the correct footnotes.

References: Adjusted to match the final reference list.

The authors state that the scientific conclusions are unaffected. This correction was approved by the Academic Editor. The original publication has also been updated.
